# Fracture Penis: An Analysis of 26 Cases

**DOI:** 10.1100/tsw.2006.363

**Published:** 2006-01-29

**Authors:** G.V.Soundra Pandyan, Ahmed Bakeet Zaharani, Mohammed Al Rashid

**Affiliations:** Department of Urology, Assir Central Hospital, ABHA, Saudi Arabia

**Keywords:** penile fracture, hematoma, surgery, complications

## Abstract

The aim of this study was to review the pattern of penile fracture occurrence, its clinical presentation, diagnosis, management, and outcome at our center. A retrospective analysis of 26 patients with penile fractures treated at our hospital from January 1997 to January 2005 was carried out. We noted an incidence of 3.5 cases per year, occurring more commonly in unmarried men. Of our study group, 28 episodes of penile fractures occurred in 26 patients. Hospital presentation after trauma varied from 2 h to 21 days. Masturbation was the main initiating causative factor and penile hematoma was the most common clinical finding. Nearly 81% noticed the characteristic click prior to the fracture. Clinical diagnosis was adequate in a majority of the cases. Midshaft fractures with right-sided laterality were more frequent in this series. The tear size ranged from 0.5—2.5 cm with a mean of 1.1 cm. All cases, but one, were treated by surgical repair using absorbable sutures. Out of three cases treated conservatively, two failed to respond and had to be treated surgically. False fracture with dorsal vein tear was present in two cases. Involvement of bilateral corpora was seen in one patient. Infection was the most common early complication, while pain with deviation was the late complication. In our experience, clinical findings are adequate enough to diagnose fracture penis in a majority of cases. Surgical exploration with repair of the tear is recommended both in early and delayed presentations. There was no noticeable relationship to the time of initial presentation or with the size and site of tear to the final outcome.

## INTRODUCTION

Penile fracture is one of the not-so-common urological emergencies. The first documented report of this fracture is credited to an Arab physician, Abul Kasem, in Cordoba over 1000 years ago[1]. It involved rupture of tunica albuginea of the corpora cavernosa. In an erect penis, this fascia is very thin and taut, thus prone for fracture due to undue sudden flexion forces. The reported mechanisms precipitating the factor are sexual intercourse, masturbation, or forceful penile manipulation. Globally, penile fracture is a rare, or at least a rarely reported, injury. There are geographical variations in the incidence and cause of penile fractures. Eke[1] reported 1642 cases worldwide in his 2002 review article. Over half of these cases, 745 (56%), were from the Mediterranean Moslem Region. Another large study reported 172 cases from a single center in Iran[2]. A more recent series by Khinev reported an annual frequency of 0.33–1.36/100,000 inhabitants with up to 2000 cases reported until 2004[3,9]. Patients present early with history of a sudden click or crackling sound in an erect penis followed by pain, sudden detumescence, and swelling. Clinical findings at presentation will include swelling, ecchymosis, penile deviation, and a palpable gap in the shaft. Immediate surgical exploration, evacuation of hematoma, control of bleeders, and repair of the tunical tear is the present trend in management[1,2]. Conservative therapy restricted to uncomplicated cases also has a good outcome[4]. An analysis of the clinical presentation, diagnosis, management, and outcome of 26 cases that presented to our center over the last 8 years is the purpose of this study.

## PATIENTS AND METHODS

Assir Central Hospital is a 600-bed, tertiary referral hospital affiliated to the King Khalid College of Medical Sciences. Data of all the cases of penile fracture from January 1997 to January 2005 (8 years) were reviewed from the case files and operating room registry; 26 patients had 28 episodes of penile fracture during this period. The age distribution, marital status, nationality, initiating cause of fracture, time to presentation to hospital, and presence of the characteristic click in these patients were analyzed. Next the clinical findings, diagnostic procedures adopted, treatment (surgical or conservative), operative findings, early postoperative complications, total duration of hospital stay, and any delayed complications during the OPD follow-up were studied. Details of the site of tunical defect, its size, and laterality were recorded along with any associated urethral injuries. For proper analysis, complications were grouped as early and delayed. Complications that occurred within 2 weeks postoperatively were grouped as early (wound infections and skin necrosis) and later than 1 month were grouped as delayed (erectile dysfunction, painful erection, and penile deviations). Erectile dysfunction was crudely assessed on follow-up by a questionnaire as to be good, mild to moderate, and poor erection (insufficient for intercourse). All patients were followed up a week after discharge and then every month up to 3 months. Only half of these patients had been followed up to 12 months. All patients underwent thorough clinical examination on admission. Presence of urethral bleeding and other associated injuries were investigated. Apart from routine blood tests, a coagulation profile was done for all patients. Ultrasound examination, Doppler study, and cavernosography were done in selective cases only. Most of the time, clinical diagnosis was enough to decide on the management option. Every patient underwent surgery under general anesthesia; exploration of the fracture site was carried out by a degloving subcoronal incision. The hematoma was evacuated and any bleeding vessels were ligated and the site of tunical defect located, measured, and then repaired by using synthetic, absorbable, inverted knot sutures. We routinely used three zero PDS sutures for all our cases. After repair, artificial erection was induced to make sure there was no leakage. A Foleys catheter 16F was inserted and retained for 2 days postoperatively. All cases received antibiotics for 5 days postoperatively with sedation for 24–48 h. None of the patients received any antierectile medication. Conservative treatment was in the form of cold compress with anti-inflammatory drugs along with antibiotic coverage. SPSS 9.0 for Windows was used to for analysis of the data.

## RESULTS

The analysis revealed that 26 cases of fracture penis occurred over a period of 8 years with an incidence of 3.5 cases per year; 28 episodes of fracture penis occurred in 26 patients. Two of the patients with the second fracture had past episodes 6 and 8 years ago and it had occurred on the same side. Details of precipitating cause, site, and size was not available. Mean patient age (Table 1) at injury was 33 years old (range 13–60). The majority of patients were Saudis (88%) compared to non-Saudis (12%). It was more frequent (50%) in the unmarried than in the married group (42%). The marital status of two patients was not known. Time from trauma to hospital presentation varied from 2 h to 21 days. Of them, 39% presented

**TABLE 1**
**Patient Characteristics and Clinical Features**

| | | |
|---|---|---|
| **Age** | 13–60 years, n (26) | Mean 32.6, SD 13.5 |
| **Nationality** | Saudi | Non-Saudi |
| n (26) | 23 (88%) | 3 (12%) |
| **Marital status** | **(n)** | **(%)** |
| Unmarried | 13 | 50 |
| Married | 11 | 42 |
| Unknown status | 2 | 8 |
| **Cause of trauma** | | |
| Coitus | 5 | 19 |
| Masturbation | 11 | 42 |
| Sudden forced flexion | 9 | 35 |
| Direct blunt trauma | 1 | 4 |
| **Clinical finding** | | |
| Hematoma | 16 | 62 |
| Hematoma + deviation | 5 | 19 |
| Ecchymosis + hematoma | 4 | 15 |
| Swelling + nodule | 1 | 4 |
| **Click( crackling sound)** | | |
| Present | 21 | 81 |
| Absent | 5 | 19 |
| **Time from trauma to presentation** | 2–504 h | Mean 69.35, SD 145.9 |
| | **(n)** | **(%)** |
| 2–6 h | 10 | 39 |
| 7–12 h | 6 | 23 |
| 13–24 h | 2 | 8 |
| 24–48 h | 4 | 15 |
| 7–21 days | 4 | 15 |

within 6 h and 30% presented within 24 h, and 31% presented late, ranging from 2–21 days. In most of the patients, the common precipitating cause for fracture was masturbation (42%), followed next by forceful flexion of the erect penis due to variety of causes like rolling over in bed, forced detumescence, and falling over. Coitus as a cause was reported by only 19% of the patients. Another unusual cause was fracture caused by direct blunt trauma to an erect penis by a heavy object. This occurred to a laborer while he was at work. He did not report to the hospital for 3 weeks because he was filled with shame; that to, because he was going on vacation to his country where he had two wives whom he had to manage! The characteristic click or crackle was present in 81% (20/26) of our patients. This was followed by detumescence and swelling. The most common clinical findings were hematoma at the site of fracture (62%), hematoma with deviation (19%), ecchymosis with hematoma (15%), and swelling with a nodule in one case. Clinical findings were enough to diagnose and decide on management in the majority of our cases. Ultrasonography was done in two cases, but was not diagnostic, it was only collaborative. Cavernosography done in one case showed clearly the defect at the fracture site with extravasation. Urethrogram or cystoscopy was not warranted in any of our cases as none of them had associated urethral bleeding or extension of the tear into the urethra. Our treatment was mainly surgical in all cases. Conservative treatment was tried in three cases because they came late (2–3 weeks); this trail of conservatism was given as the patients claimed that the swelling was regressing. The precipitating cause in these three patients was direct trauma in one, forceful flexion of the erect penis in another, and coitus in another. Two of them underwent exploration later as the swelling got worse. We did not find any undue problem in dissection and identification of the tunical defect in these delayed cases. The organized clots found at the site of fracture had to be forcefully cleared by vigorous flushing before repairing the defects in these cases. One case, however, responded well to conservative treatment and did well without any delayed complication even after a year. Of the other two delayed cases managed surgically, one had painful erection with penile deviation that had to be managed later by plication. Of the cases (Table 2) that underwent surgical exploration, we noticed that 50% were having right corporal involvement and the frequent site of tear was in the midshaft. The next common site was the proximal shaft. The mean defect size was 1.1 cm (range 0.5–2.5). One of the patients had a tear dorsally, involving both the corpora. All these tunical tears were transverse. Two of the cases revealed only dorsal vein tear with hematoma on exploration (false fracture). Hospital stay ranged to an average of 7 days (range 3–31) for all patients. The least stay was for 2 days and 77% of them stayed for less than 8 days. Wound infection was seen in four (15%) patients as an early complication (Table 3). One patient developed skin necrosis near the corona. All infections were cured with antibiotics and the case of skin necrosis was managed by split skin graft after 2 months. Delayed complications noted were pain with deviation in three patients and deviation alone in one patient. Two of the patients complained of moderate erectile dysfunction, but managed with their sexual activities without any medication. Lateral plication had to be carried out in one patient who had persistent deviation. This was in one of the patients who came as late as 3 weeks.

**TABLE 2**
**Operative Findings**

| | | |
|---|---|---|
| **Size of tear** | 0.5–2.5cm | Mean 1.108, SD 0.532 |
| **Site of tear** | **(n)** | **(%)** |
| Left corpora cavernosa | 9 | 35 |
| Proximal | 4 | 15 |
| Mid | 4 | 15 |
| Distal | 1 | 4 |
| Right corpora cavernosa | 13 | 50 |
| Proximal | 4 | 15 |
| Mid | 7 | 27 |
| Distal | 2 | 8 |
| Both corpora dorsal mid | 1 | 4 |
| Dorsal vein tear | 2 | 8 |
| Unknown (conservative Rx) | 1 | 4 |

**TABLE 3**
**Hospital Stay and Complications**

| | | |
|---|---|---|
| **Hospital stay** (n = 26) | 3–31 days | Mean 7.08, SD 5.64 |
| | **(n)** | **(%)** |
| 3–8 days | 20 | 77 |
| 10–15 days | 5 | 19 |
| 15–31 days | 1 | 04 |
| **Early complications** (<14 days) | 5 | 19 |
| Infection | 4 | 15 |
| Skin necrosis | 1 | 4 |
| **Delayed complications** (1–12 months) | 4 | 15 |
| Pain + deviation | 3 | 12 |
| Deviation | 1 | 4 |
| **Erectile dysfunction** | 2 | 8 |
| Good | — | |
| Mild to moderate | 2 | |
| Poor | — | |

## DISCUSSION

Fracture of the penis “faux pas” occurs in an erect penis and is primarily rupture of the corpus cavernosum. The tunica albuginea is among the toughest of body fascias, it measures 2 mm in a flaccid penis and gets thinned out to 0.25 mm during erection. Erection converts the safe flaccid penis into a vulnerable organ[1]. Structural anomalies of the tunica albuginea like fibrosclerosis and pholgistic cellular infiltration were found in 83% of penile fracture cases by De Rose et al.[5] and they suggested that this weakening factor could be a predisposing factor for traumatic rupture of the penis.

The most common cause for fracture penis is largely related to geographical area of report. Vigorous sexual intercourse was found to be the cause in 30–50% of cases in the West. Those from the Middle East resulted mainly from penile manipulations at masturbation[1,2,6]. In our study, we also found masturbation as the prime cause in 42% of the patients. Boujnah and Rakam[7] came to a similar interpretation when they found that 61/67 fractures in their study were due to deliberate manipulation of the erect penis. The reason for this difference could be due to strict prohibition of extramarital sex in Moslem countries. The longer time it takes to reach orgasm by manipulation than by coitus makes the penis more prone to trauma. The influence on the younger generation of the satellite television channels depicting “hot” films cannot be understressed in the Middle East. We noticed that 50% of our patients were from the unmarried group.

The distinctive presenting signs are a “click”, immediate detumescence, pain, deformity, bruise, hematoma, and deviation contralateral to the lesion. The physical appearance of the fractured swollen penis as been described as an “eggplant deformity”[2], also called as the “aubergine sign”. Urethral bleeding and a difficulty to void indicates urethral involvement. The ecchymosis or bruises will be limited to the penis in the presence of Buck’s fascia integrity. Once this fascia is disrupted, it spreads to the scrotum, perineum, and suprapubic area[1]. History of sudden click, detumescence, and hematoma were the most common features in our study. Clinical evaluation is widely considered superior to or at least equal to investigations like cavernosography, ultrasonography, or MRI. Standard ultrasounds do not rule out the diagnosis[1,2,8]. In our study, 97% of the cases were diagnosed clinically. Positive diagnosis could not be reached in two of our cases who underwent ultrasonography. Cavernosography is advocated by some authors to decide on the type of management. The tunical defect with extravasation was clearly seen in one of our cases. We do not recommend it routinely, as it is an invasive procedure and complications like priapism have been reported[9]. In two of our cases diagnosed clinically, we found dorsal vein tear without any tunical defect on exploration. Maybe cavernosography, if done in these patients, would have changed management options. These cases of so-called false fractures are recommended in the literature to be managed as any other case of penile fracture[10]. MRI is considered as the best imaging test, although it is still quite unaffordable to most.

The preponderance of right corporal fractures that has been reported by other authors is also consistent in this study. The frequency of proximal shaft fractures has been explained by the proximal location of the fulcrum of the erect penis[1,2], but we noticed midshaft fractures to be more common. Could it be explained by masturbation being the main precipitating cause, rather than coitus in comparison with other studies? There was one case with bilateral corporal tear, but none with urethral injury in this series. El–Sherif et al.[6] also had a low incidence of urethral injuries and attributed it to a high incidence of noncoital injuries like ours. Time from trauma to presentation varied in our study: 31% of the patients presented late, ranging from 2–21 days. In spite of that, 7/8 of these patients were also managed surgically. Conservative treatment was initially tried in all three of these patients, but two had to resort back to surgery as there was no improvement. We reviewed the literature on delayed surgical management of fracture penis. We found no reference on penile fractures of more than 3 weeks being treated by surgery. In one study, three cases that were 2–3 weeks old were reported, but all of them were treated conservatively[8]. Delayed surgical exploration of three cases 7–12 days old was published in another[11].

Conservative treatment of fracture penis is now an exception rather than the rule, as its results are less satisfactory than those of immediate surgical repair[1,2,4]. The World Health Organization recommends that all acute injuries to the tunica albuginea be repaired immediately by surgical intervention[12]. Synthetic, absorbable, inverted knot sutures can be used successfully for repair; nonabsorbable sutures may cause painful, palpable suture knots and should be avoided[13]. Antierectile medication after surgery is not needed, as postoperative pain during erection will prevent erection[14]. A routine Foleys catheter inserted in all our cases postoperatively also helped to hinder erection. The hospital stay ranged to an average of 7 days, which is longer compared to other studies. This is attributed to a larger percentage of cases presenting late, needing more effective treatment under observation. For the same reason, wound infection was seen as an early complication in 15% of our cases. Another unusual complication in one patient was distal skin necrosis near the corona. It had to be treated by split skin graft after 2 months. The absence of associated urethral injuries in this series is obvious when compared to other published series. The reason again is presumed to be due to the prime causative factor being masturbation rather than coitus. The additional extent of rotational axial forces during coitus may contribute to increased incidence of urethral injuries in them[1]. Delayed complications noticed were pain with deviation seen in 12% of the patients. The reported incidence of complication rates of conservative treatment is 10–53%[15] and that after immediate surgical treatment is 10%[2,14]. Being a retrospective study, the limiting factors in this series are the crude, nonstandardized questionnaire adopted for evaluating the erectile dysfunction and the lack of long-term follow-up more than a year.

In conclusion, clinical findings were adequate enough to diagnose fracture penis in the majority of cases. Noncoital cause was the common predisposing factor. The fractures were more frequent in the right corpora. Similar results were noted with other studies in the region[1,2,6,16]. Remarkable aspects are the increased frequency of tears in the midshaft and surgical management of 15% of cases presenting later than 2 weeks with good outcome. The obvious absence of associated urethral involvement and severe erectile dysfunction is noteworthy. There was no relationship between the site, size, time of repair, and the development of complications, and no difficulty in dissecting the penis in delayed cases. Our results are consistent with the literature that show the overall superiority of immediate surgery compared to conservative treatment[8,16].
